# Managing medicines in the time of COVID-19: implications for community-dwelling people with dementia

**DOI:** 10.1007/s11096-020-01116-y

**Published:** 2020-08-16

**Authors:** Heather E. Barry, Carmel M. Hughes

**Affiliations:** Primary Care Research Group, School of Pharmacy, Medical Biology Centre, Queen’s University Belfast, 97 Lisburn Road, Belfast, BT9 7BL UK

**Keywords:** Carers, COVID-19, Dementia, Independent living, Medication review, Medicines management, Primary health care

## Abstract

COVID-19 has changed life beyond recognition for millions of individuals, as countries implement social distancing measures to prevent disease transmission. For certain patient groups, such as community-dwelling older people with dementia (PwD), these restrictions may have far-reaching consequences. Medicines management may be adversely affected and deserves careful thought. PwD face unique challenges with medicines management compared to other older people, often relying upon support from family/carers and primary healthcare professionals. This article considers potential issues that PwD may face with each component of medicines management (prescribing, dispensing, administration, adherence, review), and based on previous research, highlights strategies to support PwD and their carers during this time. Primary healthcare professionals must be attentive to medicines-related needs of community-dwelling PwD, particularly those living alone, both during the pandemic and as restrictions are lifted. Carers of PwD continue to have a critical role to play in medicines management, and also require support.

## Impacts on practice

- Primary healthcare professionals must be especially vigilant to medicines-related issues in people with dementia, and particularly those living alone, during the COVID-19 pandemic.
- While remote methods of consultation and service delivery may be a useful alternative to face-to-face contact, such technologies may not always be suited to people with dementia.
- Carers have a crucial role in supporting patients and maintaining good communication with primary healthcare professionals.

## Introduction

The novel coronavirus 2019 (COVID-19) pandemic has had an international impact. While the virus appears to be receding in some areas of the world, other countries are experiencing a resurgence of cases [1]. Government and public health response is focused on slowing disease transmission, reducing hospitalisations and mortality, and preventing healthcare systems from collapsing under increased pressure. In addition to infection prevention and control measures (e.g. practising appropriate hand hygiene and respiratory etiquette), many governments have imposed stringent containment measures to restrict social contacts [2, 3]. A range of public health guidelines have been implemented, removed and reintroduced as the number of cases have increased, declined and risen again in various parts of the world. During excursions outside the home, people must maintain a distance of two metres from one another and avoid unnecessary travel [2, 3].

Older age and presence of comorbidities, such as cardiovascular disease and chronic lung disease, are known risk factors for mortality following infection with COVID-19 [4]. Specific guidance has been issued for these vulnerable patient groups. Whilst age limits for ‘older people’ may differ slightly between countries (60/65/70 years of age), in essence the advice is similar [2, 3]. In the United Kingdom (UK) for example, older people aged ≥ 70 years have been advised to be fastidious in their adherence to social distancing advice. In addition, certain sub-groups may be regarded as ‘clinically extremely vulnerable’ if they have serious, complex conditions (e.g. those who are immunocompromised and those with chronic obstructive pulmonary disease), and these groups have been instructed by their general practitioner (GP) to ‘shield’ (i.e. not leave their homes and minimise contact with other members of their households) for at least 12 weeks [2]. This could result in some older patient groups being confined to their homes for significantly longer than other members of the public. Care home residents are especially vulnerable to COVID-19, however this population is outside the remit of this article.

## Community-dwelling people with dementia and COVID-19

Whilst current restrictions and social distancing measures are a crucial intervention, for the community-dwelling older population, and particularly those with complex long-term conditions, such restrictions may have a detrimental effect on myriad facets of daily life. One patient group of particular interest is people with dementia (PwD). It is vital that PwD and their carers adhere to containment guidance; dementia is a frequently observed comorbidity amongst COVID-19 patients admitted to hospital and mortality risk has been shown to be higher among those with dementia [5, 6]. The majority of PwD live in the community, and it is estimated that one-third live alone [7]. Disruption of daily routines may be distressing and disorientating for PwD, and separation from family members and friends outside the household may add an extra layer of anguish. For those living with a spouse or other family member(s), the increased burden placed upon carers during this time should not be underestimated.

The issue of medicines management for PwD raises complex and unique challenges for both patients and carers [8, 9], and has received limited attention within the scientific literature in the past [10]. We would propose that the current restrictions may only further complicate this. Our previous research has shown that community pharmacists frequently encounter community-dwelling PwD and their carers in their clinical practice, dealing with a range of medicines-related and pharmaceutical care issues [11]. However, ways of working within primary care have changed dramatically during the current pandemic, with opportunities for face-to-face patient contact now diminished. PwD and their carers may feel that their usual support networks such as their GP or local community pharmacist are no longer as accessible to them, and they may not wish to trouble them with what they perceive to be insignificant queries during this crisis. Whilst patient contact, triage and treatment have been recommended to be delivered via telephone, email or online consultation technology where possible [12], some older PwD and their carers may be uncomfortable using these seemingly detached methods of communication and patients’ capacity to share in the decision-making process may be difficult to ascertain. This could potentially impact upon management of patients’ cognitive and non-cognitive symptoms, other comorbidities, and medicines they have been prescribed.

## Medicines management issues for people with dementia during the COVID-19 pandemic

Medicines management is multifaceted, and includes core components of prescribing, dispensing, administration, adherence, and medication review. Our recent research has focused on optimising medicines management for PwD in primary care, and we have undertaken in-depth qualitative work with patients, carers, GPs and community pharmacists to fully understand their experiences [13, 14]. We have considered each component of medicines management in terms of potential issues that may arise for PwD during the COVID-19 pandemic (Table 1), and highlighted considerations to support PwD and their carers with medicines management currently.

**Table 1:** A summary of the potential issues that may arise for people with dementia (PwD) regarding each key component of medicines management

Component of medicines management	Potential issues that may arise during the COVID-19 pandemic
Prescribing	Continued repeat prescribing of medicines that may require review for assessment of appropriateness/efficacy Non-urgent consultations in secondary care suspended, resulting in delayed treatment of dementia symptoms or other comorbidities
Dispensing	Usual face-to-face contact between community pharmacist and PwD and/or their carers diminished, reducing the opportunity for discussion about medicines management and counselling
Administration and adherence	PwD who associate medicine-taking with particular part of their daily routine that may now have changed Carer becomes unwell and/or unable to visit PwD regularly Patients’ over- or under-adherence may not be detected by GP or community pharmacist due to reduced patient contact
Medication review	Medication reviews and annual reviews for older people considered lower priority and may be suspended/deferred

*GP* general practitioner, *PwD* people with dementia

### Prescribing and review

Prescribing of regular medicines will continue in the same way and use of electronic prescribing systems (where available) will be especially useful during this time. However, one issue that could arise is the continued repeat prescribing of medicines requiring review for assessment of appropriateness and/or efficacy. Understandably, non-urgent activities, such as face-to-face medication review and annual review of older patients, are considered low priority currently [15] and are likely to be deferred until the pandemic ends. However, other members of the multidisciplinary primary care team, such as nurse practitioners or practice-based pharmacists could undertake review of medicines when dealing with repeat prescription requests and could even undertake more comprehensive medication review with patients and carers remotely. A review such as this could ensure that medication regimens are simplified for PwD to reduce the risk of medication-related harm and administration errors [16]. There has been a similar scaling back of non-urgent activity in secondary care, with many outpatient appointments either postponed (which could result in treatment delays for PwD) or taking place by telephone instead. Whilst telemedicine looks promising in terms of increased access and patient satisfaction [17], not all screening instruments used to assess cognitive function may lend themselves to be administered remotely [18, 19] which may affect assessment of drug-induced cognitive impairment. In addition, deterioration in a patient’s functional capacity, which may impact upon their ability to manage medicines, may not be detected and the patient may not self-report such difficulties. Carers have a pivotal role to play here, and should be vigilant for signs of cognitive or functional decline as well as poor control of long-term conditions, so that these issues can be raised with the patient’s GP or geriatrician. In addition, clinicians should actively enquire about and address medicines management in any consultation (either face-to-face or remote) they have with PwD and their carers.

### Dispensing

Medicines supply is unlikely to be an issue for the majority of patients. Nearly all of the PwD we interviewed had good support mechanisms in place to assist with medicines management, such as prescription ordering and acquisition undertaken by their local community pharmacy, medicines dispensed in a weekly compliance aid, and delivered directly to their home or collected on their behalf by a carer [13]. Arrangements such as these will continue as normal during the COVID-19 pandemic. In situations where a patient or carer managed collection of prescriptions or medicines themselves, GPs and community pharmacists should ensure that alternative arrangements are in place, such as electronic prescribing systems, telephoning or faxing prescriptions through to a patient-nominated pharmacy and engaging pharmacy home delivery services. However, the reduced amount of face-to-face contact between PwD (or carers) and their community pharmacist will reduce opportunities for discussion about medicines management and resolution of pharmaceutical care issues [20]. Our previous research showed that carers displayed positive intentions to address medicines-related issues with the patient’s GP or community pharmacist, and placed great trust in their advice [13]. Long-standing and established relationships between community pharmacists, their dementia patients and carers will greatly help in this situation, and community pharmacists should encourage PwD and/or their carers to make contact by telephone or email if they have medicine-related queries. Remote consultation methods using telephone or online services should be utilised by community pharmacists who wish to speak with PwD and their carers about medicines management issues, to conduct medication review or to provide counselling [20, 21].

### Administration and adherence

A number of different problems with medication administration and adherence may arise due to changes in the patient’s routine (affecting medicine-taking behaviour), reduced carer input if the carer becomes unwell or has to self-isolate, or reduced contact with their GP or community pharmacist. Many of the healthcare professionals we interviewed had serious concerns about adherence of PwD to their medication regimens, but were reassured when PwD had adequate carer support and when they were able to visit patients at home to assess their medication management routines [14]. However, healthcare professionals will be unable to monitor PwD in this manner, unless an urgent home visit is required in a clinical emergency. Community pharmacists have a key role to play in promoting continued adherence to medicines during the current pandemic and should be mindful of this when dispensing medication for PwD. Thorough assessment of the pharmacy patient medication record can often reveal potential issues with adherence, which could be followed up with more direct questioning via the remote consultation methods described above to identify solutions to resolve such issues. A similar approach should also be adopted by GPs and their colleagues when issuing repeat prescriptions. In our previous research, carers and family members were often the first individuals to bring adherence issues to the attention of healthcare professionals [14], and they will continue to play a crucial role during this pandemic. Healthcare professionals should encourage carers and family members of PwD to make contact with them if adherence problems are suspected.

### People with dementia living alone

A limitation of our previous work was our difficultly recruiting PwD living alone [13], and it is still unknown how these patients normally cope with medicines management. This patient group is especially vulnerable at the current time if they do not have family or friends to advocate for them or are socially isolated (e.g. those living in rural areas), and many of the considerations highlighted in this article will be pertinent to them. It has been shown that PwD living alone can experience difficulties using the telephone and other everyday technology such as mobile telephones and electronic devices [22–24], therefore remote consultation methods may not be as accessible to these patients who require support and/or adaptations. Healthcare professionals should be cognisant of this when suggesting to use these technologies. A useful approach may be to attempt a straightforward telephone call in the first instance, and this may need to be followed up with a face-to-face consultation if other methods of contact are not viable. Healthcare professionals should enquire about the social care support that patients may receive and ensure that such services are optimised during this time to provide adequate assistance with medicines management where necessary. There have been numerous reports globally of neighbourhood/local volunteer networks as well as formal volunteer schemes, such as the NHS Responder Volunteers in the United Kingdom and Spanish Red Cross, launched in response to this pandemic. Volunteers can create vital links between PwD and community pharmacists by assisting with delivery of medicines and providing support over the telephone or in person to check that patients are taking their medicines as prescribed.

## Conclusions

COVID-19 is placing extraordinary pressure on everyone, not least our healthcare systems. Much of the uncertainty as to how long this situation will last is adding to the fear and anxiety felt by the public. Now, more than ever, carers will play a crucial role in medicines management for PwD. As the situation continues, and as countries start to slowly return to normality, primary healthcare professionals must be vigilant to the needs of community-dwelling PwD, particularly those living alone and those who have specific problems with medicines management. Members of this vulnerable patient population should be considered a priority for medication review once non-urgent clinical activities resume.
